# Surgical Treatment of Typhoid Fever

**Published:** 1911-08

**Authors:** S. T. Barnett

**Affiliations:** Atlanta, Ga.


### Journal-Record of Medicine

Successor to Atlanta Medical and Surgical Journal, Established 1855, and Southern Medical Record, Established 1870.

**OWNED BY THE ATLANTA MEDICAL JOURNAL CO.**

**Published Monthly**

*Official Organ Fulton County Medical Society, State Examining Board, Presbyterian Hospital, Atlanta, Birmingham and Atlantic Railroad Surgeons' Association, Chattahoochee Valley Medical and Surgical Association, Etc.*

EDGAR G. BALLENGER., M. D., Editor.
BERNARD WOLFF, M. D., Supervising Editor.
A. W. STIRLING, M. D., C. M., D. P. H., J. S. HURT, B. Ph., M. D.
GEO. M. NILES, M. D., W. J. LOVE, M. D., (Ala.); Associate Editors.
E. W. ALLEN, Business Manager.

COLLABORATORS

Dr. W. F. WESTMORLAND, General Surgery.
F. W. McRAE, M. D., Abdominal Surgery.
H. F. HARRIS, M. D., Pathology and Bacteriology.
MICHAEL HOKE, M. D., Orthopedic Surgery.
CYRUS W. STRICKLER, M. D., Legal Medicine and Medical Legislation.
E. C. DAVIS, A. B., M. D., Obstetrics.
E. G. JONES, A. B., M. D., Gynecology.
R. T. DORSEY, Jr., B. S. M. D., Medicine.
L. M. GAINES, A. B., M. D., Internal Medicine.
J. N. LeCONTE, M. D., Disease of the Stomach and Intestines.
L. B. CLARKE, M. D., Pediatrics.
EDGAR PAULIN, M. D., Opsonic Medicine.
THEODORE TOEPEL, M. D., Mechano Therapy.
R. R. DALY, M. D., Medical Society.
A. W. STIRLING, M. D., etc., Diseases of the Eye, Ear, Nose and Throat.
BERNARD WOLFF, M. D., Diseases of the Skin.
E. G. BALLENGER, M. D., Diseases of the Genito-Urinary Organs.

VOL. LVIII. AUGUST, 1911 No. 5.

### SYMPOSIUM ON TYPHOID FEVER

### SURGICAL TREATMENT OF TYPHOID FEVER.

S. T. BARNETT, M. D., ATLANTA, GA.

The Surgical Treatment of Typhoid Fever relates entirely to a complication or complications which may occur during the progress of the disease and the continuation of which will jeopardize the patient's life, unless terminated by surgical interference.

It has no reference, under our interpretation, to such sequelae as may arise subsequent to the recovery of the typhoid condition itself. As an example: Osteomyelitis of the tibia two months after the typhoid patient is considered well.

It may be divided into two general heads: First, treatment instituted for complications due directly to the typhoid fever; second, for complications occurring coincidentally with the disease, but, nevertheless, requiring the services, if recognized, of a surgeon.

Under the first we have perforations of some portion of the alimentary canal, generally of the lower three feet of the ileum, though occurring in the cecum, appendix, colon, sigmoid flexure, and upper part of the small intestine. It might include also certain cases of perforation of the gall bladder, apparently owing its rupture to the typhoid state. Also rupture of spleen. I have, however, included all gall bladder conditions in the second division, as well as uterine hemorrhages or abortions, kidney or bladder disturbances, volvulus, intussusception, or any other bowel obstruction which might occur simultaneously with the typhoid lesion, but not necessarily due to it per se. Under this caption, of course, would fall any other complication requiring immediate Surgical aid, whether of a character like empyema or a fractured femur due to a fall in a state of delirium.

While recognizing the necessity of dealing with these latter complications, I will confine the bulk of my remarks to the first. Before dismissing them, however, I desire to point out two conditions, under which they should always be considered.

First, if possible, delay Surgical interference until the convalesence is completed; second, if necessary to interfere, do so on lines calculated to produce the least shock to the enteric condition compatible with the proper surgical technique in the case in point.

Having limited the subject of my discussion, practically, to perforations due to rupture through the intestinal coats, or ulcers occurring in the intima of that canal, I will try to show the best method of meeting the exigencies of the case. I might state, by way of parenthesis that the treatment of so-called "ulcer peritonitis," under which title Forbes Hawkes has recently written of Surgical complications, virtually belongs to my first division.

All treatment, whether surgical or medical, is generally described under conditions as nearly ideal as possible. The emergency may, and often does, seriously interfere with the ideal. Should Surgical complications arise, at best, all typhoids should preferably be cared for in a hospital capable of supplying proper operating facilities at any hour. In addition a House Staff, capable of recognizing a Surgical emergency as well as ordinary laboratory capacity. An operating force skilled in abdominal technique, for in possibly no operative procedures is the success of the work more dependent upon rapidity as well as skill, save in severe hemorrhages.

It is well for the attendant physician to secure the consent of the family for Surgical interference, if the case presents symtoms which would indicate the possibility of so grave a complication. Recently it was my fortune to be in a case, eventually requiring Surgery, when the life of the patient was almost sacrificed while the consent of the family was secured for the use of the knife, and in no condition does every minute count as much as in perforation. The onset of a sudden, severe pain in the lower right quadrant, generally accompanied with tenderness and rigidity of muscles, though not necessarily with an initial drop of the temperature should, in typhoid, be equivalent to an order for the operating room.

The preparation of the patient should be carried on without too much scrubbing, as the possibility of breaking down ulcers or scattering more widely the infection must be considered. In no condition do I consider the iodine preparation of more value than here, though the condition of the skin has to be considered. And this, despite the fact that I heartily agree with Tinker and Price in their recent article against its free use.

The anaesthetic of preference is gas, oxygen and ether, remebering that ether is a good cardiac stimulant. There may be contraindications to its use. Some advocate the routine use of local anesthesia, but the delay, the nervous and mental shock, plus the greater rigidity of the abdomen and pain in handling the mesentery, should decide for ether, allowing as it does, greater rapidity.

The greater number of all perforations occur near the cecum. The incision is therefore preferably through the right rectus, mostly in the lower quadrant of that side, though extending up into the upper quadrant and about four inches long. This in fat subjects should be extended, or if gall bladder involvement is suspected. If the sigmnoid seems implicated, another incision on that side is advisable. Too short an incision hampers good rapid work, and does not in itself increase the dangers. Cosmetic closures are not usually exhibited in the drawing room.

The cavity opened, the cecum is our guide, and this and the appendix rapidly inspected. The ileum is then followed until a perforation or ulcer peritonitis site found. In this inspection the use of a good assistant, and his eyes are of inestimable value, the operator giving attention to one side of the gut while his assistant watches the other. Before the inspection is over, the ascending colon is particularly scrutinized as well. While the coils are external they should be kept carefully covered and warm.

The perforation found, it should be closed with a double row of sutures of black silk with a round straight needle. These should be prepared in number beforehand.

The first rows hould be longitudinal of the gut and mattress. The second, longitudinal and Lembert. If the opening is too large to close in this manner, it may be effected by the use of an omental graft over the site. Or by resection, which multiplies the risk, or an artificial anus may be made. If these seem inadvisable, it may be walled off and allowed to drain directly through the wound.

All lesions repaired or strengthened and any excess of gas in the ileum allowed to escape, the toilet is begun. If the escape of fecal fluid is considerable, undoubtedly the best method is a copious flushing of the cavity with saline solution. If convinced that the infection does not extend beyond a limited area, I believe flushing is best omitted. Too rough handling of the peritoneum in attempts at cleansing, can not be too strongly condemned. Drainage is demanded practically always. The preferable drain is a large split rubber tube, fenestrated, gauze within, placed in the pelvis, if that locality should be drained, and cigarette drains outside both colons, ascending and descending, with two or three into the coils of the small intestine and near the site of the perforation. This, of course, in the worst cases. The ends of these drains should be brought out rather long in the lower edge of the wound. It is best not to use any iodoform gauze in these drains, as I have seen serious poisoning result. The wound should be closed in the upper edge by one or two silk worm gut sutures, sufficient to keep an extrusion of the abdominal contents.

A quantity of loose gauze should be placed around and flat gauze outside with scultetus bandage. The patient should be put in bed in Fowler's position, as it is important in my mind, that the drainage should be toward the pelvis, with its known less absorbing capacity. And this, despite the fact, that there is some more heart load in this posture; in fact, as soon as peritonitis is suspected, and before operation, the Fowler's position should be ordered, as has been pointed out by Murphy.

No one who has ever used this position can be but impressed by the less toxic effect upon abdominal drainage cases, when it is employed, and unless contraindicated, it should be maintained until convalescence is assured, if such is the happy sequel to one's efforts.

The after treatment of these cases is very important. Surgeons often times have a habit of delegating the after treatment of their cases to their assistant. This usually gives good results, despite the seeming neglect. In this condition every detail must be watched. It must not be lost sight either of the fact, that we now have a double condition to deal with. The one Surgical, the other Medical, and we must outline our treatment so as to give due weight to each. Stimulation must be carried on, and in fact, is usually instituted either before or during the operation. Nothing gives better results, as a rule, than salt solution, either by hypodermoclysis, intravenous injection, or high rectal enema or the combination of these. I would enter a word of caution here, though, as in other conditions against an almost drowning of the patient with salt solution. As to the amount that should be used, it must be left to the judgment of the operator, as to what the individual case requires, usually eight to sixteen ounces under the skin or in the vein, or about a pint in the rectum. Much larger amounts have been used, and successfully. This may be repeated as occasion demands. Strychnia or spartein are also useful before and after, and may be pushed to tolerance.

Food by the mouth should be witheld from one to four days though teaspoonful doses of water may be given after the nausea subsides, every twenty to thirty minutes. If given quite hot, it probably is absorbed before any great amount accumulates and relieves thirst. Rectal feeding should be instituted at once, and not over every six hours and usually better every eight hours, as the gut will hardly stand more. This should be preceded by a cleansing enema, to rid the lower bowel of fecal matter and mucus.

Of course, peptonization of this is necessary. As a rule, and especially so in the favorable cases, the dressings will be soaked in four to six hours, and the outer gauze should be changed. It should then be dressed from one to three times a day, as indicated. The drains may be gradually withdrawn from the third to the tenth day, care being taken to use the greatest gentleness in withdrawing them, as much unnecessary suffering may be avoided and trumatism to the gut prevented. Should fecal fistula develop, it usually heals in the course of time spontaneously. If not, the usual methods of stimulation of such tracts may be followed, or later the patient may be put under a secondary operation and the fistula eliminated.

